# Urinary Metabolic Profiling of Liver Fluke-Induced Cholangiocarcinoma—A Follow-Up Study

**DOI:** 10.1016/j.jceh.2022.11.012

**Published:** 2022-11-26

**Authors:** Munirah Alsaleh, Paiboon Sithithaworn, Narong Khuntikeo, Watcharin Loilome, Puangrat Yongvanit, Thomas Hughes, Thomas O'Connor, Ross H. Andrews, Christopher A. Wadsworth, Roger Williams, Larry Koomson, Isobel Jane Cox, Elaine Holmes, Simon D. Taylor-Robinson

**Affiliations:** ∗ Department of Metabolism, Digestion and Reproduction, Imperial College London, St Mary's Hospital Campus, London W2 INY, United Kingdom; † Cholangiocarcinoma Research Institute, Faculty of Medicine, Khon Kaen University, Khon Kaen 40002, Thailand; ‡ The Roger Williams Institute of Hepatology, Foundation for Liver Research, 111 Coldharbour Lane, London SE5 9NT, United Kingdom; § Faculty of Life Sciences & Medicine, King's College London, United Kingdom

**Keywords:** cholangiocarcinoma, mass spectroscopy, biomarkers, liver fluke, ANOVA, analysis of variance, BCAA, branched chain amino acids, CCA, cholangiocarcinoma, CID, collision-induced dissociation, CT, computed tomography, CV-ANOVA, ANOVA of cross-validated residuals, DDA, data-dependent acquisition, dCCA, distal cholangiocarcinoma, ESI, electrospray ionisation, ESI −, electrospray ionisation negative mode, ESI +, electro spray ionisation positive mode, iCCA, intrahepatic cholangiocarcinoma, LC-MS, liquid chromatography mass spectroscopy, MRI, magnetic resonance imaging, NMR, nuclear magnetic resonance, OPLS-DA, orthogonal projections to latent structures discriminant analysis, pCCA, perihilar cholangiocarcinoma, QC, quality control, ROC, receiver operating characteristic, RP, reverse phase, TOF, time of flight, UPLC, ultra-performance liquid chromatography

## Abstract

**Background/Aims:**

Global liquid chromatography mass spectrometry (LC-MS) profiling in a Thai population has previously identified a urinary metabolic signature in *Opisthorchis viverrini*-induced cholangiocarcinoma (CCA), primarily characterised by disturbance in acylcarnitine, bile acid, steroid, and purine metabolism. However, the detection of thousands of analytes by LC-MS in a biological sample in a single experiment potentially introduces false discovery errors. To verify these observed metabolic perturbations, a second validation dataset from the same population was profiled in a similar fashion.

**Methods:**

Reverse-phase ultra-performance liquid-chromatography mass spectrometry was utilised to acquire the global spectral profile of 98 spot urine samples (from 46 healthy volunteers and 52 CCA patients) recruited from Khon Kaen, northeast Thailand (the highest incidence of CCA globally).

**Results:**

Metabolites were differentially expressed in the urinary profiles from CCA patients. High urinary elimination of bile acids was affected by the presence of obstructive jaundice. The urine metabolome associated with non-jaundiced CCA patients showed a distinctive pattern, similar but not identical to published studies. A panel of 10 metabolites achieved a diagnostic accuracy of 93.4% and area under the curve value of 98.8% (CI = 96.3%–100%) for the presence of CCA.

**Conclusions:**

Global characterisation of the CCA urinary metabolome identified several metabolites of biological interest in this validation study. Analyses of the diagnostic utility of the discriminant metabolites showed excellent diagnostic potential. Further larger scale studies are required to confirm these findings internationally, particularly in comparison to sporadic CCA, not associated with liver fluke infestation.

Liver fluke-induced cholangiocarcinoma (CCA) is a serious health issue in the countries through which the Mekong River flows (Thailand, Myanmar, the Lao Peoples' Democratic Republic, Cambodia, and Vietnam).^1^ It has been reported that there are more than 20,000 new cases of liver fluke-induced CCA detected each year just in Thailand, where infestation with the liver fluke, *Opisthorchis viverrini*, is common through the practice of eating raw, fermented or partially cooked river fish.^2^ However, easy, reliable screening techniques do not exist and, currently, labour-intensive stool examination for worm eggs and mass ultrasound screening of village populations are being undertaken in the absence of a reliable point-of-care test to detect CCA.^3^

In a previous publication on patients from Thailand, we aimed to use metabonomic techniques to begin to address this situation, and several metabolic derangements were found to be potentially indicative of CCA urinary metabotype.^4^ We previously studied 48 Thai subjects at high risk of infection, 41 with active *O. viverrini* infection, 34 with periductal fibrosis and owing to the difficulty of sample collection, and only 14 with CCA.^4^ Purine and acylcarnitine derivatives were present at different concentrations in the urine of CCA patients, compared to levels observed in the urine of all the three non-cancer control groups, and of note, in the case of the acetylated carnitines in comparison to patients with periductal fibrosis.^4^

Our initial studies from the United Kingdom in patients with CCA of unknown aetiology (sporadic CCA) showed altered acylcarnitine, bile acid and purine levels in the urinary metabolome, distinct from hepatocellular and ovarian cancer but indistinct from pancreatic cancer or benign biliary strictures.^5^

Although small in sample size, we have previously compared the urinary metabolome from Thai liver fluke-induced CCA with sporadic CCA samples from British patients.^6^ Specific diagnostic biomarkers that distinguished the two groups were not apparent, but the metabolome from both groups showed altered levels of acylated carnitines, suggesting changes in energy metabolism in carcinogenesis, while differences between the urinary metabolomes from CCA patients of the two populations reflected differences in nutrition.^6^

Another group identified the potential diagnostic utility of urinary creatine riboside and N-acetylneuraminic acid in both patients with intrahepatic CCA and HCC.^7^ They were prompted to look for these metabolites after working on tumour tissue samples using mass spectroscopy (MS).^7^ The differences from our previous studies highlights the complexity of assessing many thousands of metabolites and also the biological variability which can be magnified by small studies.

MS is a key analytical platform for metabonomics; it offers sensitive and precise metabolite recovery from a given biospecimen. However, the simultaneous detection of thousands of analytes in a relativity small sample set can potentially introduce false positive findings despite correction for multiple testing using Bonferroni or similar tests.^8^ As a result, rigorous biomarker testing is required prior to their clinical implementation. The biomarker discovery pipeline consists of a sequence of preclinical phases, starting with an exploratory phase in a small pilot dataset (typically 10 samples) then usually followed by a verification phase on a set of 10–50 samples. Quantifying key biomarkers (usually <10) on 100–500 samples is then recommended before the final clinical validation, ideally on 500–1000 biological samples.^9^

To verify the preliminary urinary MS study on liver fluke-induced CCA in the literature,^4^ a larger sample set (n = 98) was collected over a 3-year period to validate the initial findings. A total of 52 urine specimens from patients with CCA were collected from the Specimen Bank of the Liver Fluke and Cholangiocarcinoma Research Center, Faculty of Medicine, Khon Kaen University. Healthy volunteers were selected from Khon Kaen as a control group for the study. To validate our previous work, the same metabolic profiling pipeline was implemented throughout from sample preparation to analytical procedures and statistical analyses.^4^

An important aspect of this study was to promote the transitioning from biomarker discovery to biomarker validation. We therefore aimed to assess the reproducibility of the assay in detecting metabolic alterations associated with the urine metabolome in CCA patients.

## Methodology

### Ethics

Urine samples were obtained from the Specimen Bank of the Cholangiocarcinoma Research Institute, Faculty of Medicine, Khon Kaen University, Khon Kaen Province in Thailand. The study was approved by the Ethics Committee for Human Research, Khon Kaen University (reference no. HE571283 and HE521209). Written, informed consent was obtained from each participant prior to recruitment. Ethical approval was also obtained from Imperial College London REC, London, UK (REC Reference 09/H0712/82). The study was conducted according to the principles set out in the 1975 Declaration of Helsinki.

### Sample Collection

Study samples were collected from the Isaan peoples, an ethnic community native to the northeastern region of Thailand. Raw, partially cooked and/or fermented fish dishes which are likely to contain the *O. viverrini* parasite are distinctive to their cultural cuisine. Patients with *O. viverrini*-induced CCA were recruited from the inpatient population in Srinagarind Hospital, Faculty of Medicine, Khon Kaen University, Khon Kaen, Thailand. Healthy Isaan volunteers were recruited from the staff of Srinagarind Hospital and Khon Kaen University staff and students. The healthy control population were recruited after a detailed questionnaire. Those who had a history of eating raw or fermented fish dishes, containing cyprinoid fish from the Mekong River were excluded, owing to the high risk of subclinical liver fluke infestation.

The study thus consisted of 98 spot urine samples (n = 52) from participants with CCA and (n = 46) from healthy volunteers. CCA was diagnosed by CT or MRI and further confirmed by histology after surgical operation.

### Sample Transport, Preparation, and MS Analysis

A courier service transported the samples (frozen on dry ice) from Khon Kaen University to St. Mary's Hospital, Liver Unit, London, UK. They were kept stored at −80°C until liquid chromatography mass spectroscopy (LC-MS) analysis at Imperial College London.

### Chromatographic Conditions

LC-MS conditions, spectral pre-processing, and metabolite annotation were as described in previous work.^4^

The spectral profiles of samples were acquired using an ACQUITY ultra performance liquid chromatography (UPLC) system (Waters Ltd., Elstree, U.K.), coupled to a LCT Premier mass spectrometer (Waters MS Technologies Ltd., Manchester, U.K.).^4^ Reverse phase (RP)-UPLC-MS was performed with electrospray ionisation (ESI) in both positive and negative modes.^4^ The conditions were optimised using quality control (QC) samples in terms of peak shape, reproducibility, and retention time.^4^

### Tandem Mass Spectrometry

Tandem mass spectrometry (MS/MS) analysis was performed using a quadrupole time-of-flight Premier instrument (Waters MS Technologies Ltd., Manchester, UK).^4^ Collision-induced dissociation experiments of the QC sample were performed for structural elucidation of detected ions in each ionisation mode.^4^ This was conducted subsequent to the original profiling run to save time and limit analytical variations in retention time and performance that can occur when returning to the instrument for collision-induced dissociation analysis.^4^ Two complementary tandem mass spectroscopy (MS/MS) acquisition modes were used to ensure sufficient MS/MS coverage of ions of interest, data-dependent acquisition and acquisition with no precursor ion selection, or data-independent acquisition (MS^E^).^4^

The data-dependent acquisition experiment was set to switch automatically from the MS to MS/MS mode using data-dependent criteria.^4^ It triggered MS/MS on the most abundant ions in each MS scan and provided fragments specifically attributed to the precursor ion.^4^

In MS^E^ mode, eluting peaks were subjected to both high and low collision energies in the collision cell of the mass spectrometer, with no prior precursor ion selection.^4^

### Metabolite Assignment Verification

The molecular mass, retention time and fragmentation spectrum of the discriminant features were compared against online spectral libraries such HMDB (www.hmdb.ca) and METLIN (https://metlin.scripps.edu). Metabolites were classified as either:

(a) identified compounds confirmed with an authentic standard.
(b) putatively annotated compounds (such as those based upon fragmentation pattern and/or spectral similarity with spectral databases).
(c) putatively identified to match a certain chemical class (such as those based on spectral similarity to known compounds of a chemical class); or
(d) as unknown compounds.

### Pre-processing

The raw LC-MS data files were converted to CSV format by MassLynx version 4.1 application manager (Waters Corporation, Milford, U.S.A.) and then imported into R Project version 3.1.0 (The R Foundation for Statistical Computing, 2014) for pre-processing using XCMS package version 2.14. (Bioconductor). Computational scripts written in-house were applied to (1) filter and identify peaks; (2) correct for retention time drift; (3) match peaks across samples; and (4) fill in missing peaks.

### Statistical Analysis

The spectral data matrix was imported to SIMCA-P+ version 13.0.2 (Umetrics, Umeå, Sweden) for multivariate statistical analysis and feature selection. Univariate significance tests and correlation analysis were performed in R Project version 3.1.0 (The R Foundation for Statistical Computing, 2014).

MetaboAnalyst v3.0 platform (http://www.metaboanalyst.ca/) was used to assess the clinical utility potential of the biomarkers.^9^ The receiver operating characteristic (ROC) curve and the area under the curve (AUC) were calculated for individual markers and for possible marker combinations. In the univariate mode, the discrimination potential of each feature was assessed by testing all possible cut-off values within all data points. The sensitivity and specificity of each marker was then calculated at the best cut-off value.

The multivariate testing of marker combinations produced ROC curves, based on the cross-validation performance of the random forest algorithm. Subsequently, the software produced a plot illustrating the order of the features, based on the frequency of being selected, which showed the stability of the rank of the metabolites. The confidence bounds of the AUCs were measured, based on re-sampling approach, using bootstrapping for the univariate AUCs or Monte Carlo cross-validation for the multi-panel biomarker analysis.^9^

All statistical analyses were initially carried out without exclusion of any metabolites and then repeated to exclude drug metabolites which were found to be present in the study population.

## Results

### Demographics, Clinical Data, and Cohort Description

A total of 100 urine samples were acquired using global LC-MS metabolic profiling. Two samples were found to be misidentified upon receiving the subsequent clinical information. One participant had ampullary carcinoma, whereas the medical condition of the second individual was not specified. Spectra from both individuals were excluded from the subsequent statistical modelling processes. Patients with CCA were notably older than the healthy group (mean age = 59 years *vs.* 31 years, respectively). Healthy volunteers were comprised mostly of women with approximately 74%, compared to 46.3% women in the CCA group.

The majority of CCA patients presented with tumours in the intrahepatic (44%) or perihilar (40%) bile ducts (Table 1). Only 15% of the CCA cohort had distal tumours. Approximately half of the cases (46.1%) presented with obstructive jaundice, particularly among patients with perihilar CCA (pCCA) and distal CCA (dCCA) tumours. None of the patients with intrahepatic tumours (iCCA) was jaundiced.

**Table 1:** Demographics of Study Population.

Characteristic	Tumour site				Healthy
	All cases	iCCA	pCCA	dCCA	
Participants, n	52	23	21	8	46
Age, mean, (range), y	59.0 (35–76)	59.7 (38–68)	**59.0 (37–76)**	55.4 (35–72)	31.1 (22–62)
Male, %	53.7	47.8	**57.1**	62.5	26.1
Serum biochemistry, mean, (range)					
*Alanine aminotransferase, (U/L)*	57.4 (7–529)	30.2 (7–105)	67.1 (14–163)	119.4 (20–529)	–
*Aspartate aminotransferase, (U/L)*	73.6 (13–>700)	37.8 (13–110)	82.5 (23–311)	164.9 (32–700)	–
*Alkaline phosphatase, (U/L)*	324 (49–1124)	144.6 (49–367)	295.6 (74–1124)	321.2 (63–843)	–
*Biliary obstruction* (jaundice %)	46.1	0	85.7	75	0

dCCA, ductal cholangiocarcinoma; iCCA, intrahepatic cholangiocarcinoma; pCCA, perihilar cholangiocarcinoma.

### Overview of MS Results from Study Samples

**Figure 1:**
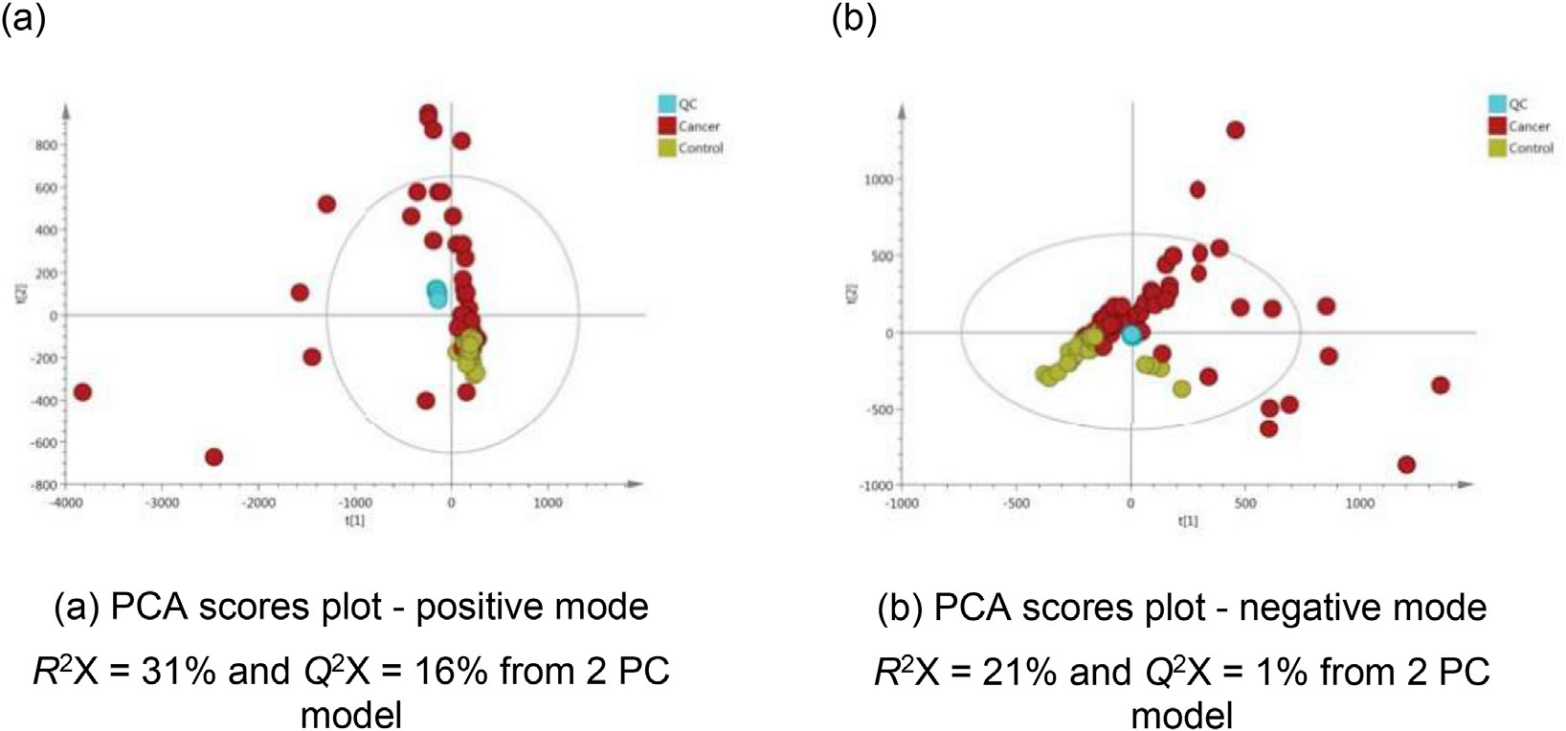
PCA scores plots of test samples and quality control (QC) samples derived from UPLC-MS urinary data (*n* = 98 participants and *n* = 11 QCs) for (a) positive and (b) negative ion mode.

Good QC clustering trends in the middle of the PCA plots indicated instrument reproducibility, but CCA samples (in red) were scattered with several outlier samples outside the 95% confidence interval in both ESI modes (Figure 1). Upon examining their raw spectral profiles, several features attributed to drug intake were present in high concentrations (Table 2).

**Table 2:** Differential Metabolites Between Cholangiocarcinoma Patients and Controls.

*m/z*	RT	Compound	Class	Adduct	Mass Error	VIP
396.044	3.86	Ceftriaxone	Antibiotic	Fragment	1	6.2
172.07	2.89	Metronidazole	Antibiotic	M+H	1	6.1
555.054	3.86	Ceftriaxone	Antibiotic	M+H	1	6.1
128.045	2.89	Metronidazole	Antibiotic	Fragment	1	5.0
105.033	3.80	Hippurate	Gut microbiota metabolite	Fragment	2	4.4
362.151	3.98	Ofloxacin	Antibiotic	M+H	0	4.0
459.274	4.20	Prednisolone tebutate	Steroid	M+H	0	4.0
152.071	2.42	Paracetamol	Analgesic	M+H	2	3.9
289.227	5.01	Bupivacaine	Local anaesthetic	M+H	1	3.7
503.306	4.31	Unknown Drug				3.7

*m/z*, mass-charge ratio; RT, retention time; VIP, variable importance in projection score.

Nine out of the top ten features that discriminated CCA patients from healthy controls using OPLS-DA were related to drug intake, predominantly antibiotics (Ceftriaxone, Metronidazole and Ofloxacin). Only one metabolite, hippurate, was endogenous and was found greater in the urine metabolome from the healthy group, Table 2. Features of drug and drug co-metabolites were excluded using correlation analysis between each feature and the metabolic data matrix.

### Cancer Patients Versus Healthy Volunteers

**Figure 2:**
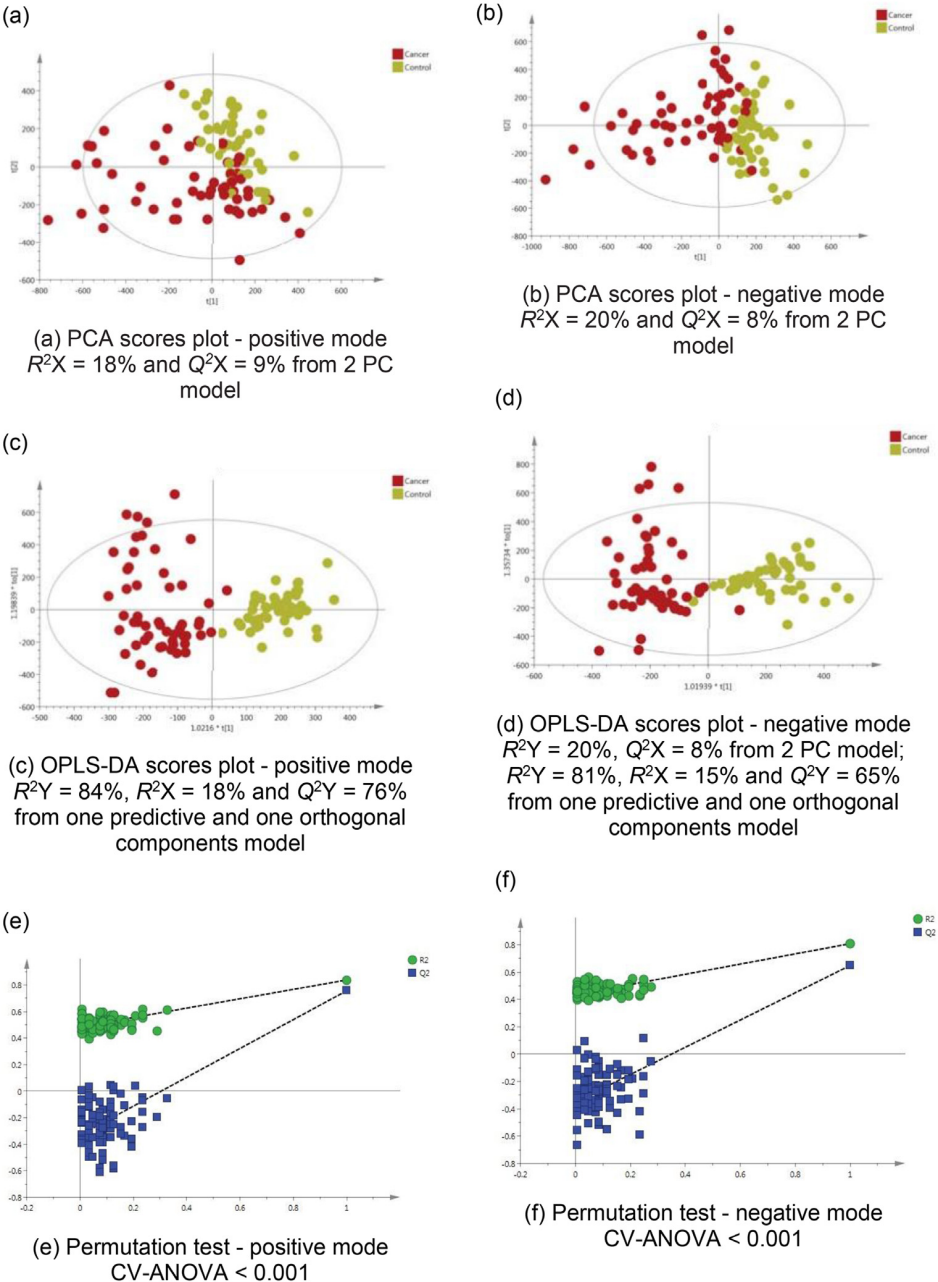
PCA scores plots for (a) positive and (b) negative ion mode data of all CCA patients and healthy volunteers. OPLS-DA scores plots showing group separation for both (c) positive and (d) negative ion mode data and the corresponding permutation tests for (e) positive and (f) negative ion mode data.

The PCA model of the urine metabolic profiles of healthy Thai participants compared to CCA patients showed clustering trend between the two groups in both ESI modes, Figure 2a,b. The OPLS-DA statistics showed enhanced separation in both modes with excellent reproducibility (Figure 2c, positive mode *Q*^*2*^ Y = 76% and Figure 2d, negative mode *Q*^*2*^ Y = 65%). The permutation test and CV-ANOVA *P*-value (<0.001) confirmed the model robustness, Figure 2e,f.

### Impact of Tumour Location on the Metabolic Profile

To address the impact of the anatomical location of CCA tumours on the endogenous metabolite profile, OPLS-DA models were computed using the spectral data from iCCA, pCCA, and dCCA. All models projected poor classification, particularly between perihilar and distal tumours (ESI positive, *Q*^*2*^Y = −85%, *P* = 1.0 and ESI negative: *Q*^*2*^Y = −58%, *P* = 1.0), indicating a very similar urine metabolome. Perihilar and intrahepatic tumours also showed relatively indistinguishable molecular profiles, based on the model statistics in positive (*Q*^*2*^ Y = 4% and *P* = 0.770) and negative ionisation mode (*Q*^*2*^ Y = 3% and *P* = 0.832).

### Cholangiocarcinoma Patients with Jaundice Versus Non-jaundiced Patients

**Figure 3:**
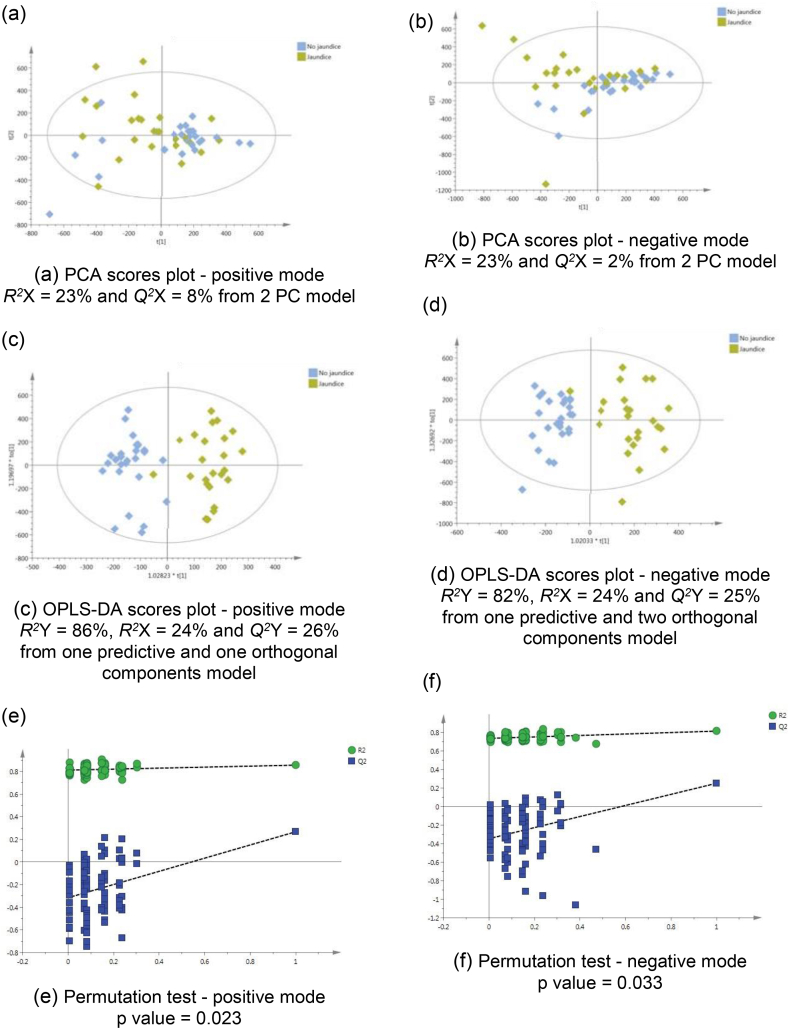
PCA scores plots for (a) positive and (b) negative ion mode data of CCA patients with and without jaundice. OPLS-DA scores plots showing group separation for both (c) positive and (d) negative ion mode data and the corresponding permutation tests for (e) positive and (f) negative ion mode data.

To assess the impact of obstructive jaundice, a supervised model was calculated using the spectral data from jaundiced CCA patients, compared to non-jaundiced CCA patients (Figure 3). The model was discriminant in both ESI modes (positive *Q*^*2*^ Y = 26% and negative *Q*^*2*^ Y = 25%) (Figure 3c,d). The CV-ANOVA *P*-value and the permutation test indicated reliable metabolic difference between the two groups, Figure 3.

The discriminant metabolites generated from the OPLS-DA model of CCA patients with jaundice, compared to non-jaundiced CCA patients are listed in Table 3. A difference in urinary bile acid levels was most pronounced in patients with obstructive jaundice. For instance, the mean relative abundance (to total signal intensity) of urinary glycocholic acid was 68, 580, and 2356 in healthy, non-jaundiced, and jaundiced CCA patients, respectively. Bile acids were significantly altered between jaundiced and non-jaundiced patients. An increase in hydroxylated acylcarnitine (C9-OH and C10:2-OH) and in vanilpyruvate, and a decrease in isocitrate, was associated with the urine metabotype of jaundiced-CCA participants.

**Table 3:** The Metabolic Proflle Altered in Cholangiocarcinoma Patients With Obstructive Jaundice.

*m/z*	RT	Metabolite	Adduct	Trend in jaundice	VIP	*P* value	FC	Identity^a^
114.066	0.51	Creatinine	M+H	D	2.5	0.851	−1.05	a
132.077	0.53	Creatine	M+H	D	2.4	0.659	−1.51	b
144.102	0.58	Proline betaine	M+H	D	2.6	0.577	−1.30	b
191.018	0.91	Isocitrate	M-H	D	4.4	0.034	−1.60	b
191.017	1.02	Citrate	M-H	D	1.8	0.680	−1.30	a
232.154	2.80	Butyryl-l-carnitine (C4)	M+H	D	2.3	0.271	−1.25	a
211.058	3.29	Vanilpyruvate	M+H	I	3.5	<0.0001	2.56	b
318.191	3.78	Acylcarnitine (C9-OH)	M+H	I	4.6	0.019	1.97	c
178.05	3.80	Hippurate	M-H	D	2.8	0.808	−1.23	a
212.001	3.81	Indoxylsulphate	M-H	D	10	0.437	−1.24	a
243.032	5.31	4-phenylbutanic acid-O-sulphate	M-H	D	2.8	0.424	−10.7	b
514.284	5.91	Taurocholic acid	M-H	I	4.1	0.008	75.0	b
544.258	5.91	Glycocholic acid sulphate	M-H	I	3.5	<0.0001	7.66	b
328.248	6.13	Acylcarnitine (C10:2-OH)	M+H	I	3.6	0.478	1.42	c
624.338	6.14	Glycochenodeoxycholic acid 3-glucuronide	M-H	I	2.8	<0.0001	12.5	b
464.299	6.23	Glycocholic acid	M-H	I	2.9	<0.0001	4.07	a
466.316	6.24	Glycocholic acid	M+H	I	3.8	<0.0001	4.52	a
528.262	6.33	Glycochenodeoxycholate-N-sulphate	M-H	I	7.4	0.0002	10.4	b
528.262	6.50	Glycochenodeoxycholate-N-sulphate	M-H	I	7.1	<0.0001	4.72	b

FC, fold change; RT, retention time; VIP, variable importance in projection score.

D, decreased; I, increased.

a Level of metabolite identification: (a) Identified compound; (b) putatively annotated compound; and (c) putatively-characterised compound class.

### Non-jaundiced Cholangiocarcinoma Patients Versus Healthy Controls

**Figure 4:**
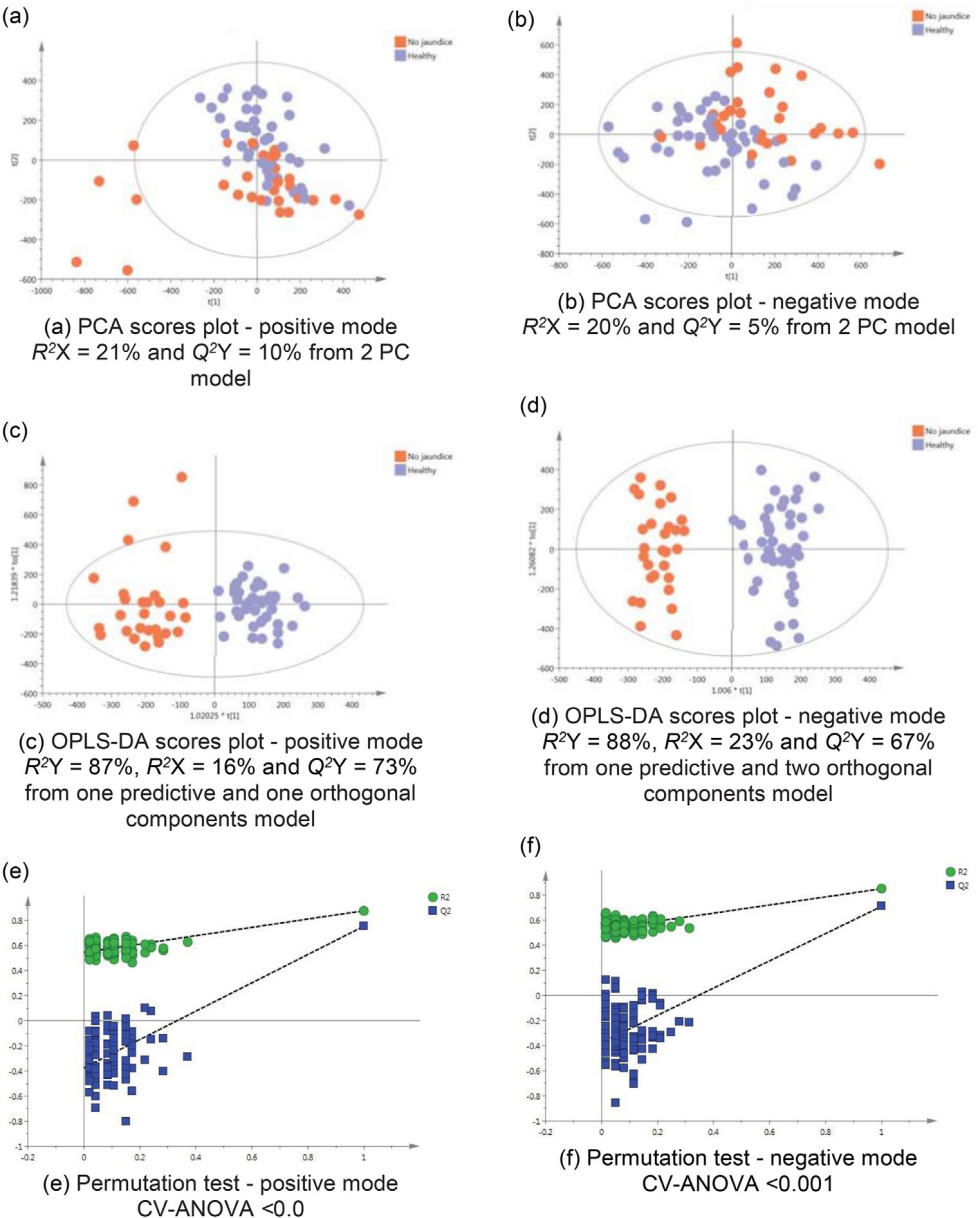
PCA scores plots for (a) positive and (b) negative ion mode data of non-jaundiced cholangiocarcinoma patients and healthy controls. OPLS-DA scores plots showing group separation for both (c) positive and (d) negative ion mode data and the corresponding permutation tests for (e) positive and (f) negative ion mode data.

The difference between the metabotype of non-jaundiced CCA patients in comparison to healthy participants was examined, Figure 4. The supervised model was highly discriminant with *Q*^*2*^Y = 77% and *Q*^*2*^ Y = 71% in the positive and negative ESI modes, respectively. The differential features between for non-jaundiced CCA patients and healthy participants are summarised in Table 4. Disturbances in isocitrate, citrate, and acylcarnitine metabolism were prominent. The leading 29 metabolites are shown in the table and whether the trends are upregulated or downregulated, compared to the control population.

**Table 4:** The Metabolic Profle Altered in Non-jaundiced Cholangiocarcinoma Patients Compared to Controls.

*m/z*	RT	Metabolite	Adduct	Trend in CCA	VIP	*P* value	FC	Identification^a^
144.102	0.58	Proline betaine	M+H	I	5.9	0.006	2.74	b
96.958	0.60	Sulphate	M-H	I	3.0	0.996	1.02	b
191.018	0.91	Isocitrate	M-H	D	3.3	<0.0001	−1.29	b
229.154	0.97	Leucyl-proline	M+H	I	3.7	0.043	1.45	b
191.017	1.02	Citrate	M-H	D	3.0	0.294	−1.36	a
166.072	1.11	7-Methylguanine	M+H	I	2.7	0.040	1.30	b
120.08	2.48	Phenylalanine^b^	+	D	4.3	0.878	−0.92	b
188.985	3.04	Pyrocatechol sulphate	M-H	D	4.1	0.014	−2.06	b
318.191	3.78	Acylcarnitine (C9-OH)	M+H	I	2.3	0.166	2.24	c
105.033	3.80	Hippurate^b^	+	D	6.1	0.0003	−1.87	a
178.05	3.80	Hippurate	M-H	D	7.8	0.001	−1.96	a
212.001	3.81	Indoxylsulphate	M-H	I	8.4	0.843	1.38	a
263.102	3.83	Phenylacetylglutamine	M-H	I	2.9	0.213	1.25	b
332.207	4.16	Acylcarnitine (C10-OH)	M+H	I	2.5	0.004	3.13	c
293.147	4.91	Unknown	M+H	D	3.0	<0.0001	−1.91	b
286.201	4.94	Acylcarnitine (C8:1)	M+H	D	5.7	<0.0001	−2.91	c
310.201	5.21	Acylcarnitine (C10:3)	M+H	D	5.4	<0.0001	−3.45	c
300.217	5.24	Unidentified acylcarnitine	M+H	D	3.3	0.162	−1.49	c
539.248	5.25	Tetrahydroaldosterone-3-glucuronide	M-H	D	2.0	<0.0001	−1.67	b
243.032	5.31	4-phenylbutanic acid-O-sulphate	M-H	I	4.4	0.350	7.31	b
383.152	5.39	3b,16a-Dihydroxyandrostenone sulphate	M-H	D	2.7	<0.0001	−4.03	b
302.233	5.56	2,6 dimethylheptanoyl carnitine (C9:0)	M+H	D	3.0	0.013	−1.77	b
312.217	5.65	2-trans,4-cis-decadienoylcarnitine (C10:2)	M+H	D	2.6	<0.0001	−3.75	b
314.233	5.80	Acylcarnitine (C10:1)	M+H	D	3.9	<0.0001	−2.02	c
331.174	5.98	17ɲ-hydroxyprogesterone	M-H	D	3.1	0.066	−1.54	b
367.157	6.33	Steroid sulphate (C^19^ H^28^ O^5^ S)	M-H	D	2.6	0.041	−3.53	c
465.248	6.37	Steroid glucuronide (C^25^ H^38^ O^8^)	M-H	D	2.3	<0.0001	−1.97	c
528.262	6.50	Glycochenodeoxycholate-N-sulphate	M-H	I	3.1	0.297	2.82	d
495.295	6.58	Pregnanediol-3-glucuronide	M-H	D	2.2	0.002	−4.20	b

FC, fold change; RT, retention time; VIP, variable importance in projection score; CCA, cholangiocarcinoma

D, decreased; I, increased.

a Level of metabolite identification: (a) Identified compound; (b) putatively annotated compound; (c) putatively characterised compound class; and (d) Unknown.

b *m*/*z* represents a fragment of this metabolite.

### The Diagnostic Performance of Candidate Biomarkers

Individual metabolites of interest are shown in Table 5. Of note, citrate and isocitrate were decreased in CCA patients with acylcarnitine (C9-OH) and acylcarnitine (C10-OH) were increased in the patient population, compared to the ethnically matched Isaan healthy volunteers. AUROC curves for different metabolite panels were calculated. The AUC values of potential biomarker combinations yielded high values ranging from 0.942 using only two metabolites to 0.99 using 20 or 25 molecules. The best diagnostic accuracy of 93.4% was achieved, with a total of 10 metabolic features.

**Table 5:** The Areas Under the Curve (AUC) Values of Individual Urinary Markers.

Metabolite	Trend in CCA	AUC (%)	95% CI	Cut-off	Sensitivity (%)	Specificity (%)
**Acylcarnitine:**						
2-trans,4-cis-decadienoylcarnitine (C10:2)	D	95.2	89.2–99.0	425	92.9	91.3
Acylcarnitine, (C10:3)	D	94.4	88.6–98.1	1160	85.7	91.3
Acylcarnitine, (C8:1)	D	85.9	76.3–93.2	2240	78.6	80.4
Acylcarnitine, (C10-OH)	I	84.4	75.2–91.9	614	75.0	78.3
Acylcarnitine, (C10:1)	D	83.1	72.0–91.7	1880	71.4	84.8
2,6 dimethylheptanoyl carnitine, (C9:0)	D	74.0	62.1–85.6	1720	60.7	89.1
Acylcarnitine, (C9-OH)	I	68.6	56.2–80.3	822	64.3	65.2
Unidentified acylcarnitine, 300.217	D	70.9	56.4–82.8	2650	76.1	67.9
**Steroids**:						
3b,16a-Dihydroxyandrostenone sulphate	D	88.4	79.9–88.8	775	84.8	85.7
Steroid glucuronide (C25 H38 O8)	D	86.4	75.8–94.6	1810	84.9	85.7
Tetrahydroaldosterone-3-glucuronide	D	83.5	72.9–91.7	1390	80.4	78.6
Pregnanediol-3-glucuronide	D	78.9	67.5–88.6	414	60.9	85.7
Steroid sulphate (C19 H28 O5 S)	D	78.4	65.8–88.7	496	69.6	75.0
17ɲ-hydroxyprogesterone	D	63.5	51.1–75.3	5230	67.9	56.5
**Other:**						
Glycochenodeoxycholate-N-sulphate	I	85.5	75.1–94.3	464	78.6	82.6
Hippurate	D	74.7	62.6–84.3	18800	71.4	67.4
7-Methylguanine	I	72.9	59.0–83.6	5050	64.3	76.1
Pyrocatechol sulphate	D	70.8	58.3–82.5	3150	53.6	78.3
Leucyl-Proline	I	70.1	57.0–81.9	6290	63.0	78.6
Isocitrate	D	65.1	50.7–77.9	11500	71.4	58.7
4-phenylbutanic acid-O-sulphate	I	62.8	48.4–76.3	77	50.0	80.4
Phenylacetylglutamine	I	61.6	47.5–73.9	19000	50.0	80.3
Proline betaine	I	61.8	45.4–75.1	5390	53.6	89.1
Phenylalanine	D	60.3	45.6–75.5	4390	57.1	73.9
Indoxylsulphate	I	60.1	44.6–74.9	35700	57.1	58.7
Sulphate	I	52.6	31.8–61.3	3810	50.0	58.7
Citrate	D	52.4	39.1–67.6	4810	64.3	58.7

AUC, area under the receiver operating characteristic curve; CI, confidence intervals; CCA, cholangiocarcinoma.

D, decreased; I, increased.

## Discussion

A total of 98 spot urine samples were used to characterise liver fluke-induced CCA signatures using a MS-based metabonomics approach. Clinical information including the anatomical location of the tumours and the presence of obstructive jaundice were used to stratify the patients and explore the effect on the detected metabolite profiles. The presence of obstructive jaundice had a clear impact on bile acid urinary elimination, whereas no obvious difference was observed, based on the tumour anatomical location. Acylcarnitine perturbation was pronounced in urine metabolic profile from CCA patients. Environmental and physiological factors, such as drug intake, were found to be the most influential confounding factors.

Despite its promising potential, metabolic profiling holds some intrinsic flaws. The biological and experimental limitations associated with such a system biology approach can challenge its biological interpretation and clinical implementation. Various factors can be a source of variability in the MS data matrix produced using the training and validation samples, which are generally divided into 1) pre-analytical and 2) instrumental or analytical-related.^8^

Pre-analytical causes of variability in the detected metabolic profiles can affect the sample at any point from the sample selection and collection until the sample preparation for chromatographic analysis. The stability of human urine specimens can be potentially jeopardised because of sample-related factors, such as prolonged storage and freeze thaw cycles.^10^ Additionally, physiological factors, including age, gender, ethnicity and disease statues, and environmental factors (such as diet, drugs, physical activity, diurnal cycles, and the host-gut microbiota metabolic interactions) can increase inter-individual biological diversity and mask the true biomarker associations relative to the disease by the addition of noise.^8^ For example, the presence of drug-related analytes in a sample can affect the presence of metabolic features, as a result of sample saturation and can potentially challenge the interpretation of the results.

It is therefore critical to study these non-disease-specific factors which can significantly induce a high level of noise in the urinary metabolic profiles. The pre-analytical variability can be partially controlled for by matching the participants on demographic characteristics (such as age and gender), collection of sufficient metadata, and standardising the dietary recording and sample handling protocols.

Sources of technical variability can be accounted for by standardising the analytical pipeline, including the use of the same instrument, column type, solvent batch and instrument parameters (such as mass resolution and collision energy). Small variations between MS experiments in retention times and accurate mass measurements are expected. Furthermore, it is also possible to experience variation across the same analytical run such as retention time drift over the run. Therefore, intermittent injection of QC samples is routinely used to assess the analytical variation in the MS system.^8^

Nevertheless, it is difficult to assess precisely what influences the reproducibility and selectivity of untargeted MS signal detection. Ion suppression and/or ion-enhancement due to the endogenous compounds within the sample (such as salts, proteins and lipids) and the presence of xenobiotic substances (such as drugs) are named “matrix effects”. The suppression or enhancement response of an analyte results when an analyte present in a sample, other than the analyte of interest compete to be ionised. The volatility of compounds in a sample is another factor that can impact the number of ions detected.^11^ Furthermore, the degree of droplet evaporation in the ESI source alters the amount of charged ions in the gas phase.^11^

Evidence of perturbation in bile acid homoeostasis and increased urinary elimination of excess bile acids in patients with obstructive jaundice have been acknowledged in the late 1960s.^12^ In the current study, a dramatic increase in urinary bile acid excretion was the most pronounced feature in jaundiced-CCA patients (fold change increase from 75 to 4), compared to non-jaundiced CCA patients. Taurine- and glycine-conjugated cholic acid (taurocholic acid, glycocholic acid), conjugated glycocholic acid and glycochenodeoxycholic were significantly increased in jaundiced-CCA patients, compared to non-jaundiced patients.

To a lesser extent, the urinary excretion of acylcarnitine species was also dysregulated in jaundiced patients. Two medium-chain hydroxylated acylcarnitines, acylcarnitine (C9-OH) and acylcarnitine (C10:2-OH), were elevated in jaundiced patients compared to the non-jaundiced group urine metabolome. Greater urinary concentrations of acylcarnitine (C9-OH) have been detected in Chinese colorectal cancer patients and a rise in the plasma level of acylcarnitine (C10:2-OH) was found implicated in recovery after exercise.^13^^,^^14^ Acylcarnitine implication in CCA with jaundice is uncertain, but it is possibly due to compromised liver capacity to oxidise fatty acid in clinically-icteric patients.^15^

A reduction in the urinary relative abundance of citrate and its isomer, isocitrate, was generally associated with CCA patients, but it was more pronounced in patients with biliary obstruction. This was contrary to the observation found in a recent Chinese LC-MS metabolic profiling study of urine samples from extrahepatic CCA patients, where urinary citrate levels were higher in cancer patients.^16^ Shao and colleagues identified general reduction in urinary citrate in presence of liver disease, both benign (cirrhosis) and malignant (HCC), but the metabolite exhibited no significant difference between the two groups.^17^ An observation which was confirmed by Chen and colleagues, where citrate was only significantly reduced in HCC patients with cirrhosis and hepatitis.^18^ The decrease in citrate excretion in urine is possibly related to TCA cycle perturbation, resulting from mitochondria dysfunction in liver disease.^19^

A compound putatively identified as vanilpyruvate, a catecholamine and phenylpyruvate derivative, was significantly associated with jaundiced patients (VIP = 3.5, *P* < 0.0001 and FC = 2.56). Vanilpyruvate is reported to be increased in the urinary metabolic profile of individuals with aromatic l-amino acid decarboxylase deficiency, a disorder that impairs the synthesis of serotonin, dopamine and catecholamines.^20^ Several other compounds were found to be differential between jaundiced and non-jaundiced CCA urine samples, but not to a level of significance. Greater abundance of creatinine, creatine, proline betaine, citrate, hippurate and indoxylsulphate was found to be associated with jaundiced patients, whereas, 4-phenylbutanic acid-O-sulphate and butyryl-l-carnitine were down-regulated, compared to the non-jaundiced group.

A multivariate statistical model was computed using the spectral data of CCA patients (excluding jaundiced participants) versus healthy controls to identify a panel of urinary metabolic markers related to CCA-genesis. Several metabolites belonging to various metabolic pathways, particularly related to acylcarnitine and steroid metabolism, were found to be perturbed. Assessment the validity of these metabolites and their relevance to hepatobiliary disease was carefully reviewed before considering these for future evaluation as potential markers in CCA.

As an example, leucyl- or isoleucyl-proline was ruled out from our diagnostic panel: the dipeptide was higher in men, and it is therefore possibly influenced by protein intake. Previous MS studies on animals have shown influence of disease and diet on leucyl-proline levels. The urinary clearance of the peptide decreased in rats with atherosclerosis.^21^ In another study, the plasma levels of leucyl-proline decreased after 7 weeks of hypercholesterolemic diet.^22^ Leucyl-proline was also significantly (*P* < 0.001) reduced in human MS urinary profiles in bladder cancer patients but was found to be higher in HCC.^23^^,^^24^ Therefore, because of the ambiguous kinetics leading to these observations, leucyl-proline was not included as one of the diagnostic markers.

Compounds classified as gut microbial co-metabolites and/or dietary phenols (hippurate, phenylacetylglutamine, indoxyl sulphate, pyrocatechol sulphate, 4-phenylbutanic acid-O-sulphate, and phenylalanine) are influenced by several factors other than cancer. For instance, hippurate excretion has been observed to be higher in females, as reviewed by Lees and co-workers.^25^

Lees and colleagues also reported reduction in hippurate urinary clearance in obesity, diabetes, inflammatory bowel disease, parasitic infection, and cancer, as observed in numerous publications investigated using a metabonomic approach.^25^ Analysis of the diagnostic power of these markers generally showed poor performance (AUC <70) and/or wide confidence intervals. These markers did not contribute greatly to the multi-panel marker classification.

The AUC values and the corresponding CIs of potential biomarker combinations yielded high values ranging from 0.942 using only two metabolites to 0.99 using 20 or 25 molecules. The best diagnostic accuracy, 93.4%, was achieved with a total of 10 metabolic features. The performance compares favourably to CA-19, the currently used clinical marker, which has been reported to have a sensitivity and specificity of 40–70% and 50–80%, respectively, in CCA patients.^26^

Acylcarnitine and steroid species yielded the best classification performance and were selected in the multipanel model. Accumulation of hydroxylacylcarnitine species (C9-OH and C10-OH) was a distinctive pattern in the CCA urine metabolome. Levels of saturated (C9) and unsaturated (C8:1, C10:1, C10:2 and C10:3) long-chain acylcarnitine species were significantly reduced in CCA, particularly in patients without obstructive jaundice. An acylcarnitine, 2-trans, 4-cis-decadienoylcarnitine (C10:2), was shown to be the most frequently selected metabolite, based on a repeated random sub-sampling cross-validation (n = 50 runs).

Steroid-related analytes also showed excellent diagnostic utility. Four steroid metabolites, tetrahydroaldosterone-3-glucuronide, 3b,16a-dihydroxyandrostenone sulphate, pregnanediol-3-glucuronide, and steroid glucuronide, were selected among the diagnostic panel. Low urinary elimination of steroids was observed in all CCA patients, regardless of jaundice. Urine steroid metabonomics studies revealed a promising diagnostic utility for distinguishing early HCC from cirrhosis and adrenocortical carcinoma from adrenocortical adenomas.^27^^,^^28^ The strong male predominance of HCC in males prompts the question on the involvement of sex hormones (particularly androgens) in hepatocarcinogenesis.^29^

The role of various agents in modulating biliary carcinogenesis has been thoroughly studied in experimental models.^30^^,^^31^ Steroid hormones have been shown to play a role in supporting the growth of the biliary epithelium.^31^ The bile epithelial network possess a number of biological functions (such as secretion, absorption, proliferation, regenerative processes, and signalling) that is regulated by several agents including neuropeptides, steroid hormones, cytokines, and growth factors.^30^^,^^32^ In the presence of cholangiopathies, the maintenance of biliary functions is greatly affected as the balance between proliferation/loss of cholangiocytes is lost.^31^

Oestrogens, for example, are well known carcinogenic agents in oestrogen-responsive tissues and have been recently shown to play a role in promoting CCA cellular growth and apoptosis.^31^ Cholangiocytes of normal liver do not express oestrogen receptors, but in presence of biliary malignancy, oestrogen receptors were positive in >80% of CCA cells.^33^ Oestrogens were found to favour the growth and proliferation of malignant mass by synergising the activities of growth factors and by taking part at both receptor and post-receptor levels.^34^ Thus, they play a critical role in inducing neo-angiogenesis in oestrogen-sensitive cancers through the activities of vascular endothelial growth factor (VEGF) receptors, which in turn mediate the proliferative effects of oestrogens.^34^ Mancinelli *et al.* suggested that CCA tumours could be consider oestrogen dependent and the use of anti-oestrogen drugs should control the growth of these tumours.^35^

Interestingly, glycochenodeoxycholate-N-sulphate was also selected among the biomarker panel. The abundance of bile acid metabolites varied within the non-obstructive CCA group, implicating those factors other than jaundice status affected bile acid homoeostasis in a subgroup of patients. Accumulation of bile acids in urine has been associated with an inflammatory signature in various forms of hepatobiliary diseases.^36^ Furthermore, dysregulation of bile acid metabolism was found to be implicated in biliary tract carcinogenesis by activating pathways that promote cholangiocellular proliferation and increases CCA invasiveness, such as the G protein-coupled bile acid receptor TGR5.^37^

One of the most significant limitations of this study was the non-availability of age-matched healthy controls without a history of raw or fermented cyprinoid fish consumption. Unfortunately, the eating of such high-risk foods has been all too prevalent for generations in the Isaan region of Thailand. Public health education programmes have only recently affected dietary behaviour in the younger generation, allowing the collection of suitable controls.^38^ We were unable to obtain age-matched healthy volunteers for our older CCA patients for this reason, but we admit that future studies should try to address this issue or use non-Isaan Thai populations where high-risk foods are not traditionally consumed.

In addition, future studies should control for drug usage, as many of the metabolites initially identified in the urinary metabolome were drug metabolites although our analyses were rerun to specifically exclude those that were identified. Previous studies have also highlighted nicotine metabolites, bacterial co-metabolites, and metabolic by-products from the diet.^4^ All of these are potential limitations. In future, larger studies should stratify for drug usage, cigarette smoking, and where possible, diet. In order to control for dietary influences, a detailed dietary questionnaire should be incorporated.

In conclusion, the study highlights several metabolic alterations in biliary carcinoma and builds on the initial pilot study, where we studied 48 Thai subjects at high risk of infection, 41 with active *O. viverrini* infection, 34 with periductal fibrosis and owing to the difficulty of sample collection, only 14 with CCA, all of whom were non-jaundiced.^4^ In the current study, pronounced bile acid urinary excretion was mostly associated with clinically icteric CCA patients. Similar to our initial study, patients without jaundice, acylcarnitine, and steroid species were the most discriminant, compared to the disease-free cohort. Targeted acylcarnitine and steroid assay could potentially be informative; it can provide a greater understanding with the benefits of higher specificity, lower potential for false positive identifications and better control for environmental confounders (such as diet and drugs). Gut microbial co-metabolites (such as hippurate and indoxyl sulphate) and bile acids have been shown to exhibit a different pattern in cancer patients. Emerging evidence has identified a distinct colonic microbial population “on” and “off” the tumour microenvironment, implicating an interplay between intestinal microbial ecology and carcinogenesis.^39^ Better understanding of the gut microbial-mammalian co-metabolism and the role of the oncogenic microbiome in disease initiation and progression may provide novel diagnostic and therapeutic tools.^40^

The rare occurrence of CCA in Western countries limits biomarkers validation in large human cohorts. Nevertheless, CCA is one of the most common malignancies in Thailand and the framework for a large-scale surveillance programme is already available. Preclinical validation of a urinary prognostic and diagnostic metabolic panel could be implemented in parallel to the exciting ultrasound screening programme known as CASCAP.^38^

Work on urinary proteomic biomarkers has already been published in this context.^41^ Lysosome-associated membrane glycoprotein 1 (LAMP1), lysosome-associated membrane glycoprotein 2 (LAMP2) and cadherin-related family member 2 (CDHR2) were found to be potential candidate biomarkers to distinguish non-malignant from malignant biliary strictures in this Thai population with liver fluke infection.^41^ It is possible that parallel metabolomic studies of soluble metabolites, such as the current line of investigation, in tandem with proteomic approaches may be a fruitful avenue to explore to develop biomarkers for early CCA detection.

## Ethics approval

Ethical approval was obtained from Khon Kaen Human Research Ethics Committee, (references HE571283 and HE521209) and from Imperial College London REC, London, UK (REC Reference 09/H0712/82).

## Consent to participate

Prior written, informed consent was obtained from each participant.

## Consent for publication

Not applicable.

## Availability of data and material

Available from Larry Koomson, Department of Metabolism, Digestion and Reproduction, Imperial College London, London, W2 INY, United Kingdom (l.koomson@imperial.ac.uk).

## Code availability (software application or custom code)

Not applicable.

## Declaration

This paper is based on a chapter in the thesis of the first author, Munich Alsaleh—to which all authors had academic contribution or supervision. Professor Puangrat Yongvanit and Professor Narong Khunikeo died during preparation of this manuscript. They contributed substantially to the work. Approval has been obtained from their legal representatives for their inclusion in this manuscript as an author. Further details are available from Professor Ross H. Andrews, Cholangiocarcinoma Research Centre, Faculty of Medicine, Khon Kaen University, Khon Kaen 40002, Thailand (rhandrews@gmail.com). Professor Roger Williams also died during manuscript preparation but contributed substantially to the work. Approval has been obtained from his legal representatives for his inclusion in this manuscript as an author. Further details are available from Natalie Day at The Institute of Hepatology, 111 Coldharbour Lane, London SE5 9NT, United Kingdom (n.day@researchinliver.org.uk). The families of all three deceased authors were happy that their names were included in the authorship as a testament to their work.

## Credit authorship contribution statement

All authors made substantial contributions to the conception and design of the work. The acquisition of data was undertaken by T.O.'C., T.H., L.K., P.S., N.K., W.L., P.Y., and C.W. Analysis was performed by M.A. with interpretation of data by I.J.C., P.S., N.K., E.H., R.H.A., and S.D.T.-R. M.A. drafted the work with input from I.J.C., R.W., E.H., and S.D.T.-R. All authors revised it critically for important intellectual content. All authors approved the version to be published and agree to be accountable for all aspects of the work in ensuring that questions related to the accuracy or integrity of any part of the work are appropriately investigated and resolved.

## Conflicts of interest

All authors have none to declare.

## Acknowledgements

All authors acknowledge the United Kingdom National Institute for Health Research Biomedical Research Centre at Imperial College London for infrastructure support. We are most grateful to Mary M.E. Crossey, Sushmita Sen, Thomas A. Barbera, Nicola A. Cook and Yasmin Pasha for help with sample collection and storage. Álvaro Del Valle Palacios helped with the production of the figures.

## Funding

This study was funded by grants from the Wellcome Trust ISSF Fund at Imperial College London and AMMF – the Cholangiocarcinoma Charity (Stansted, Essex, UK). MA was funded by the StratiGrad PhD program at Imperial College London. Running costs were also provided by a generous donation from the friends and family of Mr and Mrs Barry Winter and of Mrs Suzy Dunn.
